# A Förster Resonance Energy Transfer Switchable Fluorescent Probe With H_2_S-Activated Second Near-Infrared Emission for Bioimaging

**DOI:** 10.3389/fchem.2019.00778

**Published:** 2019-11-25

**Authors:** Rongchen Wang, Wei Gao, Jie Gao, Ge Xu, Tianli Zhu, Xianfeng Gu, Chunchang Zhao

**Affiliations:** ^1^Key Laboratory for Advanced Materials and Feringa Nobel Prize Scientist Joint Research Center, School of Chemistry and Molecular Engineering, Institute of Fine Chemicals, East China University of Science and Technology, Shanghai, China; ^2^Department of Medicinal Chemistry, School of Pharmacy, Fudan University, Shanghai, China

**Keywords:** ratiometric, light up, FRET, AIE, NIR-II imaging

## Abstract

Real-time and accurate detection of endogenous hydrogen sulfide is of great biomedical significance. Here, a FRET-based fluorescent probe for ratiometric detection of H_2_S was designed to comprise an AIE luminophore TPE as an energy donor and a monochlorinated BODIPY dye as an energy acceptor and H_2_S-responsive site. Such a designed probe showed H_2_S-dependent ratiometric and light-up NIR-II emission, enabling accurate imaging of H_2_S-rich cancer cells and identification of H_2_S-rich tumors with high resolution.

## Introduction

Endogenous hydrogen sulfide is an important signaling molecule, mainly derived from the enzymatic hydrolysis of L-cysteine (Chiku et al., 2009; Singh et al., 2009). Studies have found that H_2_S is associated with many pathological processes, while an abnormal level of H_2_S may associated with some diseases, such as Alzheimer's disease, hypertension, and cardiac ischemia disease (Eto et al., 2002; Zhao et al., 2015; Shi et al., 2017). Therefore, real-time and accurate detection of hydrogen sulfide is of great biomedical significance. Until now, many fluorescence-based H_2_S probes have been reported (Jin et al., 2016; Wang et al., 2018, 2019b); however, the fluorescence of many probes generally locates in the visible or the near-infrared I region (650–900 nm), inevitably leading to some drawbacks of poor tissue penetration, severe background interference from living tissue (Zhou et al., 2014). Compared with the traditional NIR-I imaging (650–900 nm) (Li et al., 2018), fluorescent imaging in the second near-infrared window (NIR-II, 1,000–1,700 nm) has attracted more and more attention due to lower tissue absorption, stronger tissue penetration, and reduced autofluorescence (Hong et al., 2012; Dang et al., 2016; Shi et al., 2018; Xu et al., 2018). Another issue is the hydrophobic nature of most traditional fluorescent dyes, which generally triggers the aggregation in physiological conditions due to π-π stacking. Such a process can give rise to aggregation-caused quenching (ACQ) (Sun et al., 2014; Yuan et al., 2015) and thus compromise the accuracy of bioimaging. In comparison, fluorogens with AIE characteristics show enhanced fluorescence in the aggregate states, thus providing an alternative strategy for the design of fluorescent light up probes (Zhao et al., 2012; Mei et al., 2014, 2015; Zhang et al., 2015; Fu et al., 2019). Since the hydrophobic fluorescent probes undergo the intrinsic aggregation process in aqueous media, it is desirable to develop H_2_S-activatable probes with AIE characteristic for *in vivo* imaging.

**Scheme 1:**
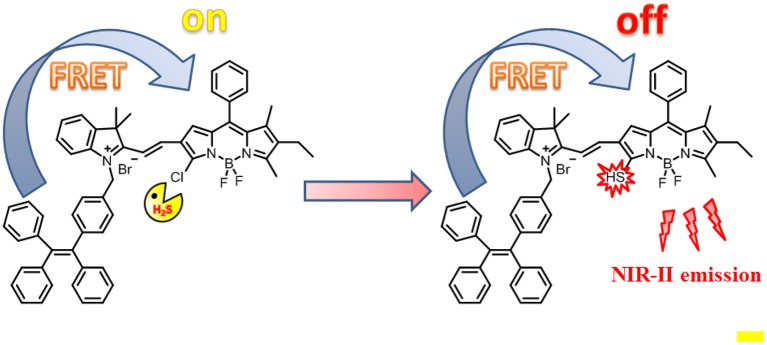
Schematic illustration of the design of probe TPE-BODIPY-Cl and the mechanism for H_2_S-mediated ratiometric and NIR fluorescence light up.

Herein, we reported a H_2_S-responsive probe that showed ratiometric fluorescence and NIR-II emission light-up upon activation for *in vitro* and *in vivo* imaging (Scheme 1). Such a probe was designed by appending an AIE luminophore TPE, as an energy donor, to a monochlorinated BODIPY dye as an energy acceptor and H_2_S-responsive site. As compared to conventional intensity-based fluorescent probes (Huang et al., 2014; Tang et al., 2016), this Förster resonance energy transfer (FRET)-based ratiometric probe can enable accurate detection through elimination of the limitations of experimental conditions including probe concentration, light source, and background interference effects (Wang et al., 2019a). Such a design strategy is applicable to the design of various ratiometric probes for different targets. As expected, in the absence of H_2_S, due to the good spectral overlap between the emission spectra of the TPE and the absorption spectra of the BODIPY, efficient FRET occurs. In contrast, in the presence of H_2_S, the absorption spectra of BODIPY undergo an obvious red shift, resulting in a significant reduction of the overlap with the TPE emission. Correspondingly, the FRET process is significantly attenuated. More importantly, upon activation by H_2_S, the probe produces a new fluorescence light-up at 920 nm with the fluorescence tail extending to 1,300 nm, indicative of the suitability for fluorescent imaging in the second near-infrared (NIR-II).

## Experimental

### Synthesis

**Scheme 2:**
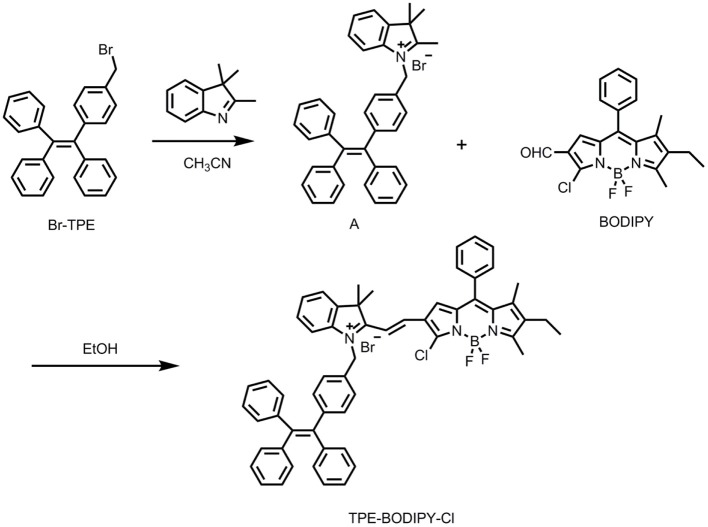
Synthesis of the target compound TPE-BODIPY-Cl.

The TPE-BODIPY-Cl was obtained from the synthetic route of Scheme 2. Br-TPE and BODIPY were synthesized according to the literature procedure (Zhao et al., 2013, 2014). Animal experiments were performed in compliance with Chinese legislation on the Use and Care of Research Animals and guidelines by Fudan University Animal Studies Committee for the Care and Use of Laboratory Animals. All experimental procedures were approved by this committee.

### Synthesis of Compound A

Br-TPE (165 mg, 0.39 mmol) and 2,3,3-trimethyl-3H-indole (62 mg, 0.39 mmol) were dissolved in 25 mL CH_3_CN and refluxed for 10 h. Then, the solvent was removed under reduced pressure, and the crude product was dissolved in CH_2_Cl_2_. The mixture was dropped into the ether solvent to precipitate white solid, which was used for next reaction without further purification. HRMS (ESI, m/z): calculated for C_38_H_34_N [M-Br]^+^: 504.2691, found: 504.2699.

### Synthesis of Compound TPE-BODIPY-Cl

Compound A (100 mg, 0.17 mmol) and BODIPY (80 mg, 0.21 mmol) were dissolved in dry ethanol and refluxed for 4 h. Then, EtOH was evaporated and the crude product was purified by a silica gel column with CH_2_Cl_2_/MeOH (20/1, v/v) as eluent to give TPE-BODIPY-Cl (90 mg, 56%). ^1^H NMR (400 MHz, CDCl_3_) δ 8.00 (d, 1H, J = 12.00 Hz), 7.63–7.55 (m, 2H), 7.52–7.46 (m, 4H), 7.43–7.36 (m, 2H), 7.09–7.06 (m, 7H), 7.03–7.00 (m, 3H), 6.98–6.96 (m, 4H), 6.95–6.92 (m, 4H), 6.87–6.85 (m, 4H), 6.08 (s, 2H), 2.71 (s, 3H), 2.44-2.39 (q, 2H, J = 6.67 Hz), 1.78 (s, 6H), 1.52 (s, 3H), 1.09–1.05 (t, 3H, J = 8.00 Hz). ^13^C NMR (101 MHz, CDCl_3_) δ 144.43, 144.21, 143.73, 143.25, 143.06, 142.64, 141.75, 141.48, 139.85, 132.12, 131.79, 131.20, 130.44, 129.50, 129.38, 129.06, 128.96, 127.76, 127.68, 127.61, 126.62, 126.52, 126.39, 122.35, 115.31, 51.90, 31.94, 29.71, 29.67, 29.37, 27.60, 22.71, 17.29, 14.14, 13.94, 13.90, 12.81. HRMS (ESI, m/z): calculated for C_58_H_50_BF_2_N_3_Cl [M-Br]^+^: 872.3754, found: 872.3750.

## Results and Discussion

TPE-BODIPY-Cl was prepared via a Knoevenagel condensation reaction. The synthesis and characterization are outlined in Scheme 2 and Supporting Information.

### Spectroscopic Studies of TPE-BODIPY-Cl

**Figure 1:**
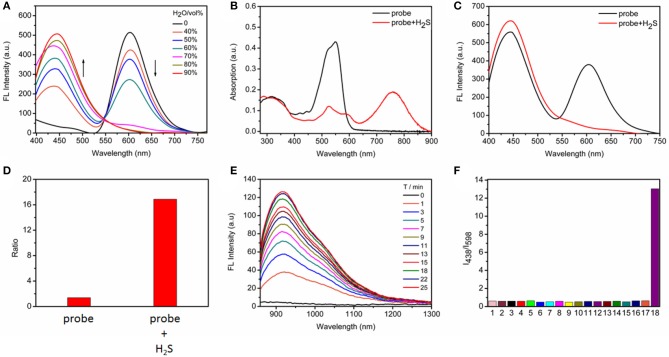
**(A)** Fluorescence changes of TPE-BODIPY-Cl (10 μM) in Tris/CH_3_CN mixtures with different water fractions. **(B)** Absorption and **(C)** fluorescence spectra in the absence and presence of 100 μM NaHS in Tris/CH_3_CN buffer solution (0.5 M Tris-HCl, 40% CH_3_CN, pH = 7.4), λ_ex_ = 360 nm. **(D)** The fluorescence intensity ratio (I_438_/I_598_) in the absence and presence of 100 μM NaHS. **(E)** Time-dependent NIR-II emission spectra upon addition of 100 μM NaHS, λ_ex_ = 760 nm. **(F)** Ratiometric fluorescence changes of TPE-BODIPY-Cl in the presence of 100 μM NaHS and other biologically relevant reactive sulfur and anions (1 mM) in Tris/CH_3_CN buffer solution (0.5 M Tris-HCl, 40% CH_3_CN, pH = 7.4): (1) Free; (2) F^−^; (3) Cl^−^; (4) Br^−^; (5) I^−^; (6) ${\text{NO}}_{2}^{-}$; (7) ${\text{N}}_{3}^{-}$; (8) ${\text{HCO}}_{3}^{-}$; (9) ${\text{SO}}_{4}^{2-}$; (10) ${\text{HPO}}_{4}^{2-}$; (11) ClO^−^; (12) H_2_O_2_; (13) ^−^OAc; (14) S_2_${\text{O}}_{3}^{2-}$; (15) GSH; (16) Cys; (17) Hcy; (18) NaHS.

With the probe in hand, we initially evaluated the photophysical properties. Because the probe contains the TPE AIEgen, we explored the AIE performance of the probe to obtain the best test conditions. As shown in Figure 1A, we tested the FRET process of the probe under different ratios of H_2_O/CH_3_CN. With the increasing water content, the degree of aggregation of the probe intensifies, accompanying the increase of TPE fluorescence while the occurrence of ACQ for BODIPY chromophore. As is well-known, a ratiometric fluorescence mode has higher accuracy than turn-on or turn-off fluorescence detection mode (Wang et al., 2015; Zhang et al., 2019), we here selected Tris/CH_3_CN buffer solution (0.5 M Tris, 40% CH_3_CN, pH = 7.4) as the next testing condition in order to obtain the ratiometric fluorescent responsiveness. The aggregation of our probe under this buffer solution was proven by dynamic light scattering (Figure S1).

Next, we evaluated the response capability of TPE-BODIPY-Cl toward H_2_S (Figure 1 and Figure S2). As shown in Figure 1B, the free probe showed strong absorption at 550 nm. The typical absorption band of the TPE around 300–360 nm was also noted. In the fluorescence spectrum, due to FRET process, we can observe two strong fluorescence peaks with maxima at 438 and 598 nm, corresponding to TPE and BODIPY, respectively. When treated with 100 μM H_2_S, the absorption band at 550 nm decreased significantly and a new absorption band appeared at 760 nm with a red-shift of 220 nm due to the formation of TPE-BODIPY-SH that was proven by HRMS analysis (Figure S3). Such treatment with NaHS attenuated the FRET, thus affording an enhancement of the fluorescence intensity ratio (I_438_/I_598_) by 12 times. This indicated that TPE-BODIPY-Cl was indeed a ratiometric fluorescent probe for H_2_S. Most importantly, H_2_S-triggered a new NIR-II fluorescence light up with a maximum emission of 920 nm (λ_ex_ = 760 nm). These results demonstrated that TPE-BODIPY-Cl could be used as a H_2_S-activatable NIR-II fluorescent probe to enable high-resolution bioimaging with deep-tissue penetration. Utilizing the linear relation of fluorescence intensity ratio at 438 and 598 nm with H_2_S concentration (0–50 μM) (Figure S4), the detection limit was determined to be 6.5 × 10^−7^ M, indicating that TPE-BODIPY-Cl has high sensitivity for H_2_S detection. Of note, the probe exhibits minimal optical responsiveness to biologically related reactive sulfur (RSS), oxygen (ROS), and nitrogen species (RNS) and some ions, showing its high selectivity for H_2_S (Figure 1F and Figure S5). In addition, the good photostability of probe TPE-BODIPY-Cl, evidenced by minimal optical changes under continuous irradiation with light irradiation (Figure S6), indicated its suitability for bioimaging.

### Imaging of H_2_S in Living Cells

**Figure 2:**
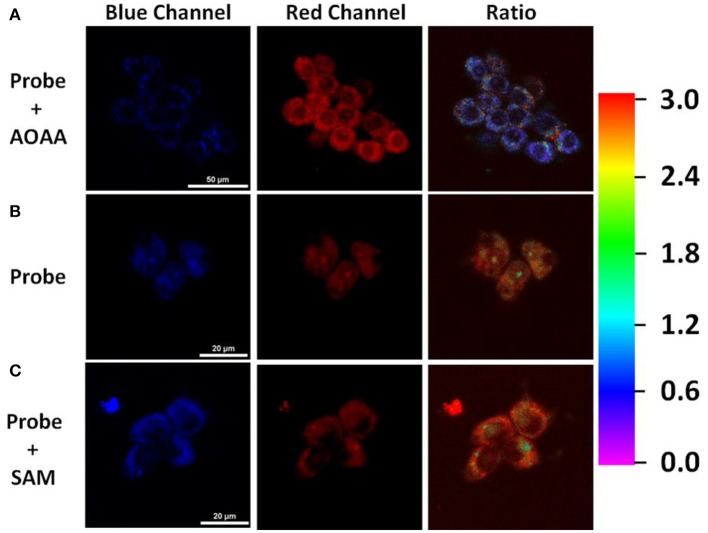
**(A)** HCT116 cells pretreated with 1 mM AOAA for 1 h, followed by incubation with TPE-BODIPY-Cl (10 μM) for 30 min. **(B)** HCT116 cells incubated with TPE-BODIPY-Cl for 30 min. **(C)** HCT116 cells pretreated with SAM (3 mM) for 1 h, followed by loading with TPE-BODIPY-Cl for 30 min.

Inspired by the promising response to H_2_S, we then assessed the ability of TPE-BODIPY-Cl for fluorescence imaging in living HCT116 cells that express high levels of H_2_S [20–100 μM, Szabo et al., 2013]. As shown in Figure 2, the incubation of HCT116 cells and 10 μM TPE-BODIPY-Cl for 30 min afforded the bright and stable fluorescence signal in the green channel and relatively weak fluorescence in the red channel. The ratio of the green to red channel is ~2.82. When a CBS inhibitor aminooxyacetic acid (AOAA) which can inhibit the H_2_S production was added, the ratio dropped to 0.70. In contrast, with the addition of an allosteric CBS activator S-adenosyl-L-methionine (SAM) to promote the production of H_2_S, the ratio increased to 3.59. These results indicated that TPE-BODIPY-Cl can efficiently enter living cells and serve as a potential sensor to detect endogenous hydrogen sulfide rapidly and specifically.

### Imaging of H_2_S *in vivo*

**Figure 3:**
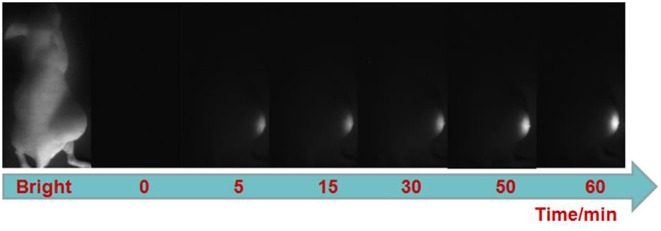
NIR-II fluorescent images of HCT116 subcutaneous xenograft nude mice. Images were taken at various time points after subcutaneous injection of TPE-BODIPY-Cl into tumor region.

Finally, we explored the ability of the probe for visualizing H_2_S-rich cancers using HCT116 subcutaneous xenograft nude mice. TPE-BODIPY-Cl was administrated to nude mice through intratumoral injection. As shown in Figure 3, after the injection, obvious NIR-II fluorescence in the tumor region was observed and the signals gradually increased over time, producing a 14.8-fold enhancement at the time point of 60 min (Figure S7). These results indicated that the TPE-BODIPY-Cl could be activation of NIR-II fluorescence in H_2_S-rich colorectal cancers.

## Conclusion

In summary, we have designed a FRET based probe through appending the AIE luminophore TPE to the monochlorinated BODIPY dye for imaging of H_2_S-rich cancer cells and tumors, wherein TPE serves as an energy donor and BODIPY dye as an energy acceptor. This probe showed H_2_S-dependent FRET process, thus enabling the selective visualization of endogenous H_2_S in HCT116 cells. Furthermore, this probe displayed H_2_S specific activation of NIR II emission light up. By using this activatable NIR II emission, accurate identification of colorectal tumors was realized. We expect our design strategy here can help the development of a new activatable probe.

## Data Availability Statement

The raw data supporting the conclusions of this manuscript will be made available by the authors, without undue reservation, to any qualified researcher.

## Ethics Statement

The animal study was reviewed and approved by Fudan University.

## Author Contributions

CZ and XG conceived the project and wrote the manuscript. RW conceived the molecule design. RW and WG prepared and characterized the small molecule. RW, TZ, and GX performed the optical characterization. RW and JG performed the living cells imaging, *in vivo* imaging, and analyzed the data.

### Conflict of Interest

The authors declare that the research was conducted in the absence of any commercial or financial relationships that could be construed as a potential conflict of interest.
