# Supplementary material for: A Förster Resonance Energy Transfer Switchable Fluorescent Probe With H2S-Activated Second Near-Infrared Emission for Bioimaging
Source: Front Chem. 2019 Nov 25;7:778. doi: 10.3389/fchem.2019.00778 (PMC6886478; doi:10.3389/fchem.2019.00778)
Supplement: Supplementary file 1 [file Table_1.DOCX]

A Förster Resonance Energy Transfer Switchable Fluorescent Probe with H_2_S-Activated Second Near-Infrared Emission for Bioimaging

Rongchen Wang,^†,§^ Wei Gao, ^†,§^ Jie Gao,^‡,§^ Ge Xu,^†^ Tianli Zhu,^†^ Xianfeng Gu,^‡,^* Chunchang Zhao^†,^*

^†^Key Laboratory for Advanced Materials and Feringa Nobel Prize Scientist Joint Research Center, Institute of Fine Chemicals, School of Chemistry and Molecular Engineering, East China University of Science and Technology, Shanghai, 200237, P. R. China.

E-mail: zhaocchang@ecust.edu.cn

^‡^Department of Medicinal Chemistry, School of Pharmacy, Fudan University, Shanghai, 201203, P. R. China.

E-mail: xfgu@fudan.edu.cn

^§^These authors contributed equally.

**1. DLS measurement.**


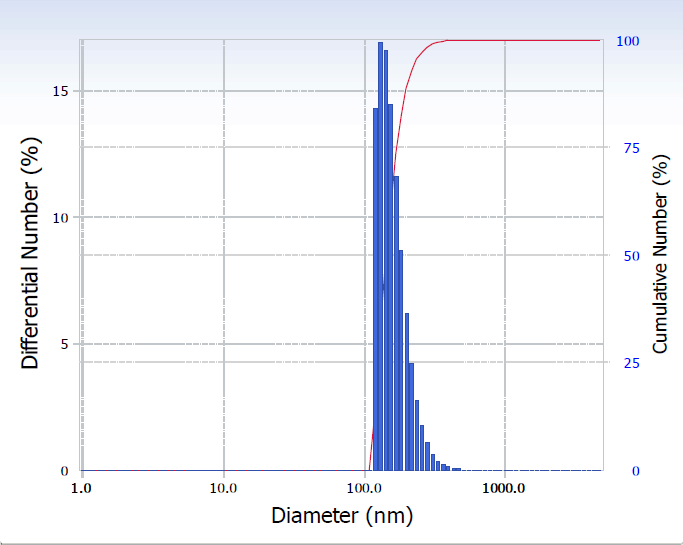


Figure S1. Dynamic light scattering measurements of TPE-BODIPY (10 μM) in Tris/CH_3_CN buffer solution (0.5 M Tris-HCl, 40% CH_3_CN, pH = 7.4).

**2. Time-dependent spectra changes of TPE-BODIPY-Cl in the presence of H_2_S.**


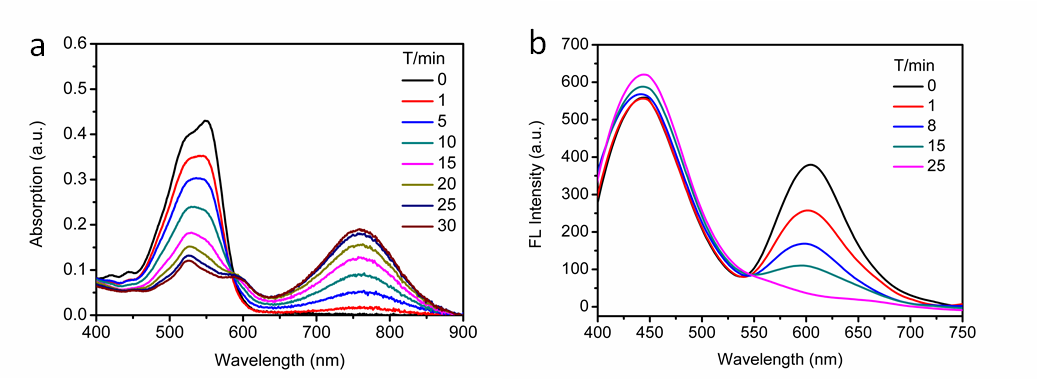


Figure S2. Time-dependent spectra changes of TPE-BODIPY-Cl (10 μM) in the presence of NaHS (100 μM). a) Absorption, b) fluorescence in Tris/CH_3_CN buffer solution (0.5 M Tris-HCl, 40% CH_3_CN, pH = 7.4), λ_ex_=360 nm.

**3. HRMS analysis.**


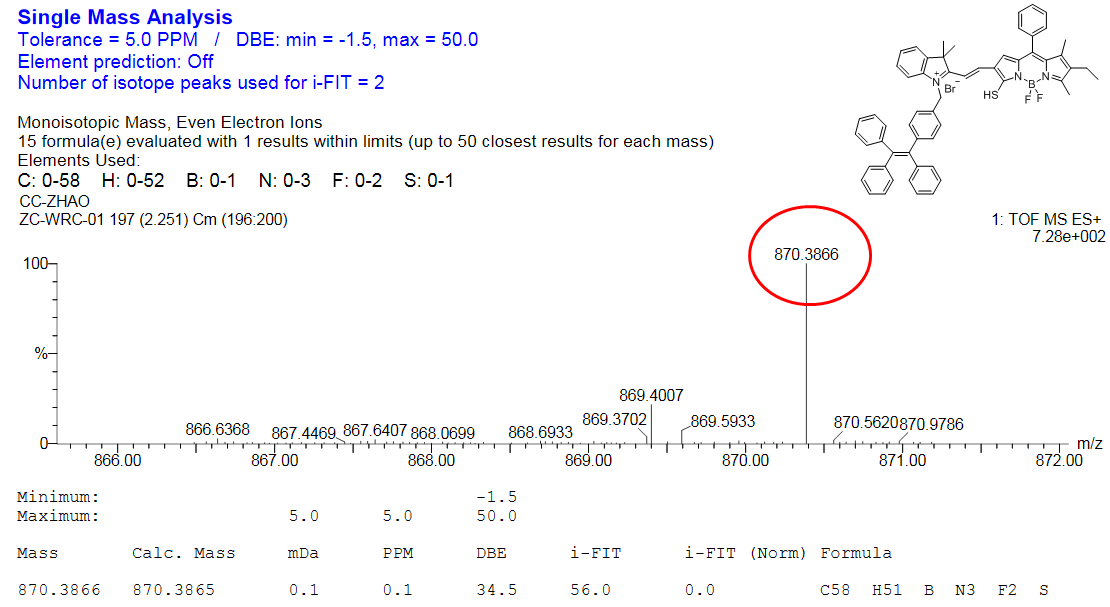


Figure S3. HRMS characterizations of the products from reactions of TPE-BODIPY-Cl + NaHS.

**4.** **Linear correlation profile.**


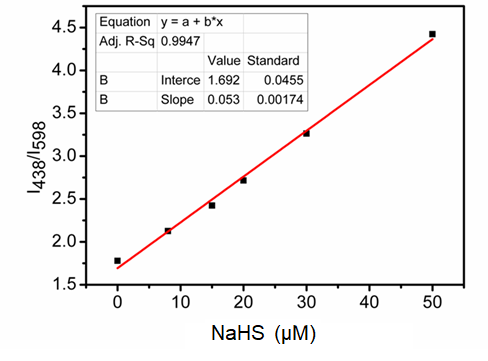


Figure S4. Linear correlation between fluorescence intensity ratio (I_438_/I_598_) changes of TPE-BODIPY-Cl and the NaHS concentration.

**5. TPE-BODIPY-Cl exhibited high specificity for H_2_S.**


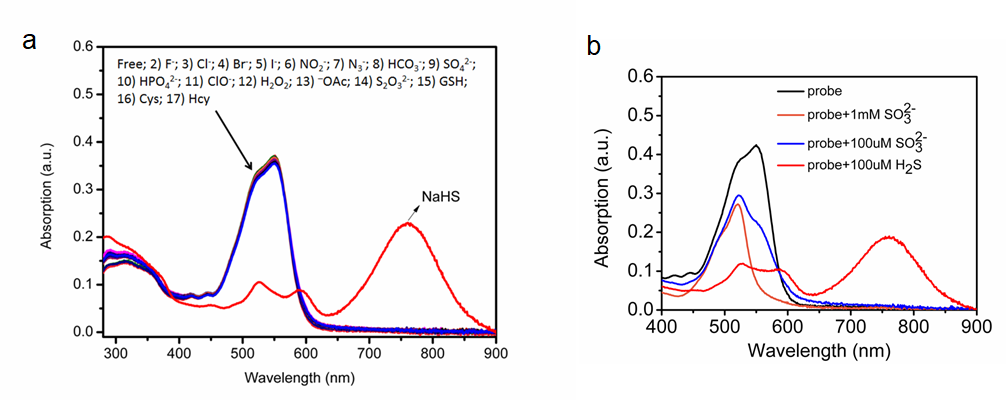


Figure S5. TPE-BODIPY-Cl exhibited high specificity for H_2_S in Tris/CH_3_CN buffer solution (0.5 M Tris-HCl, 40% CH_3_CN, pH=7.4)

**6. photostability.**


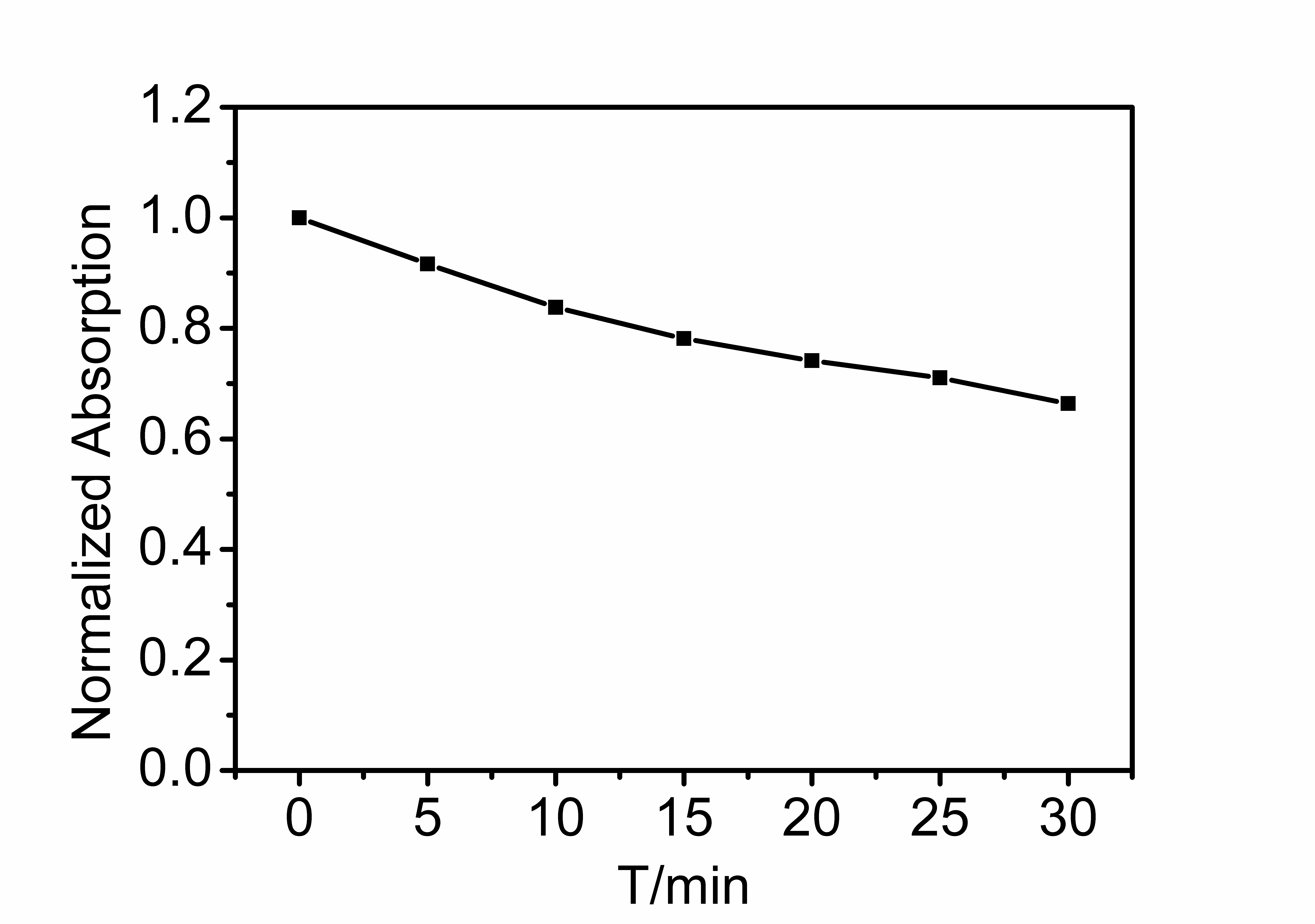


Figure S6. Time course of absorption changes (normalized) of probes in buffer under continuous irradiation at 530 nm with an LED lamp (100 mW): λ_ab_ = 550 nm.

**7. NIR-II fluorescence in tumor.**


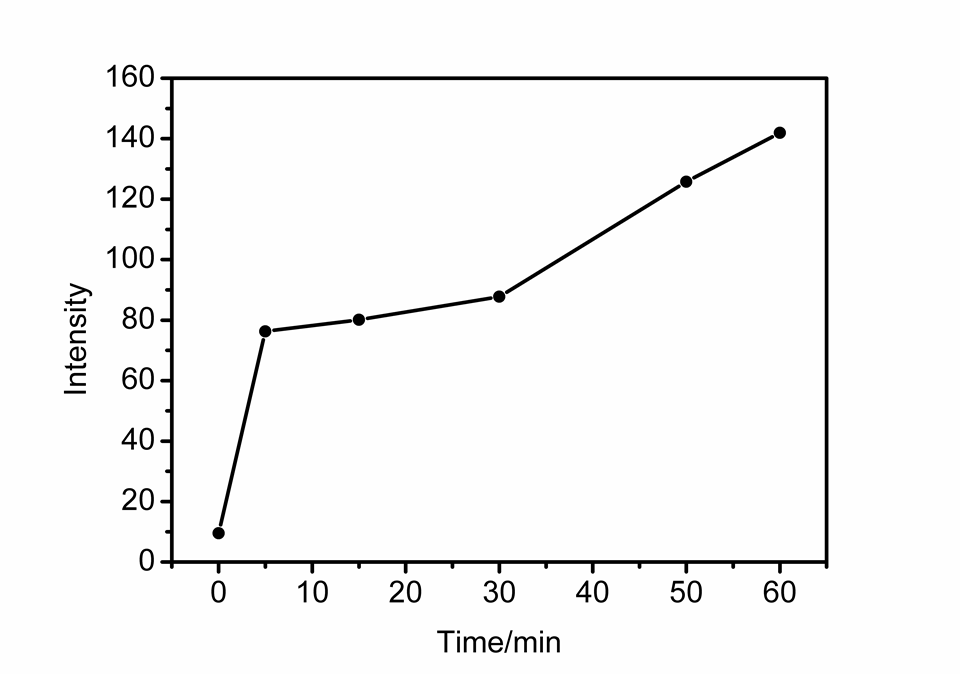


Figure S7. The changes of NIR-II fluorescence intensity at the tumor site with time.

**8. NMR and MS characterizations.**


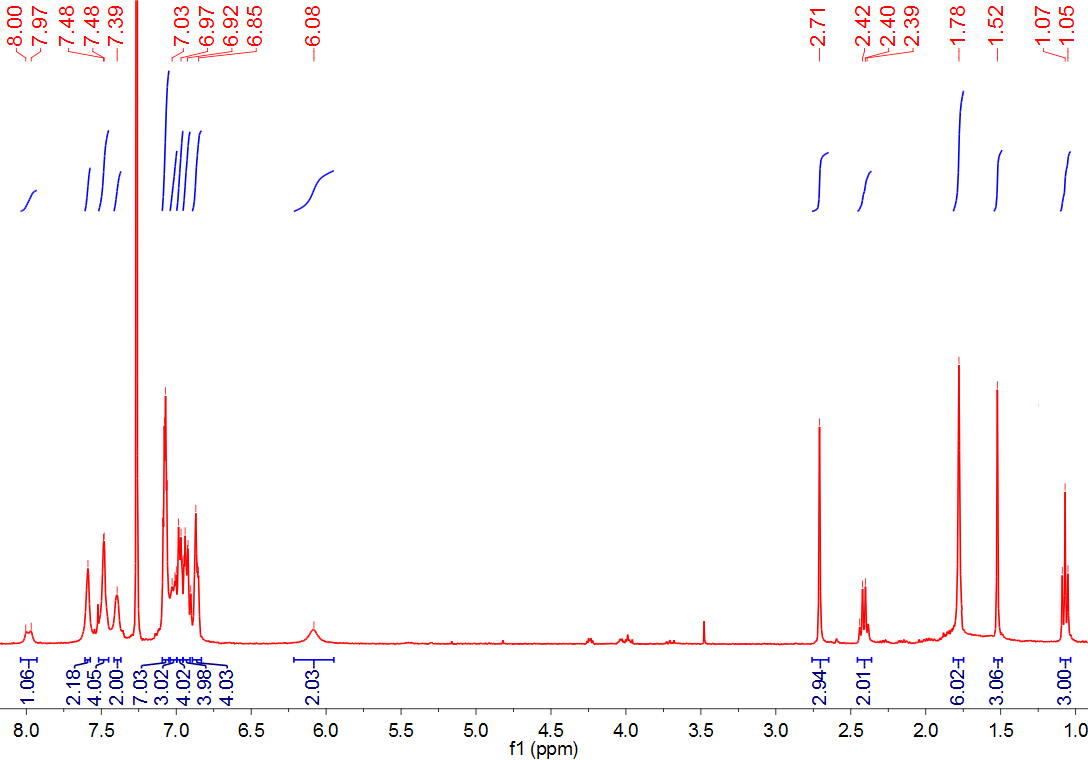


^1^H NMR spectrum for TPE-BODIPY-Cl


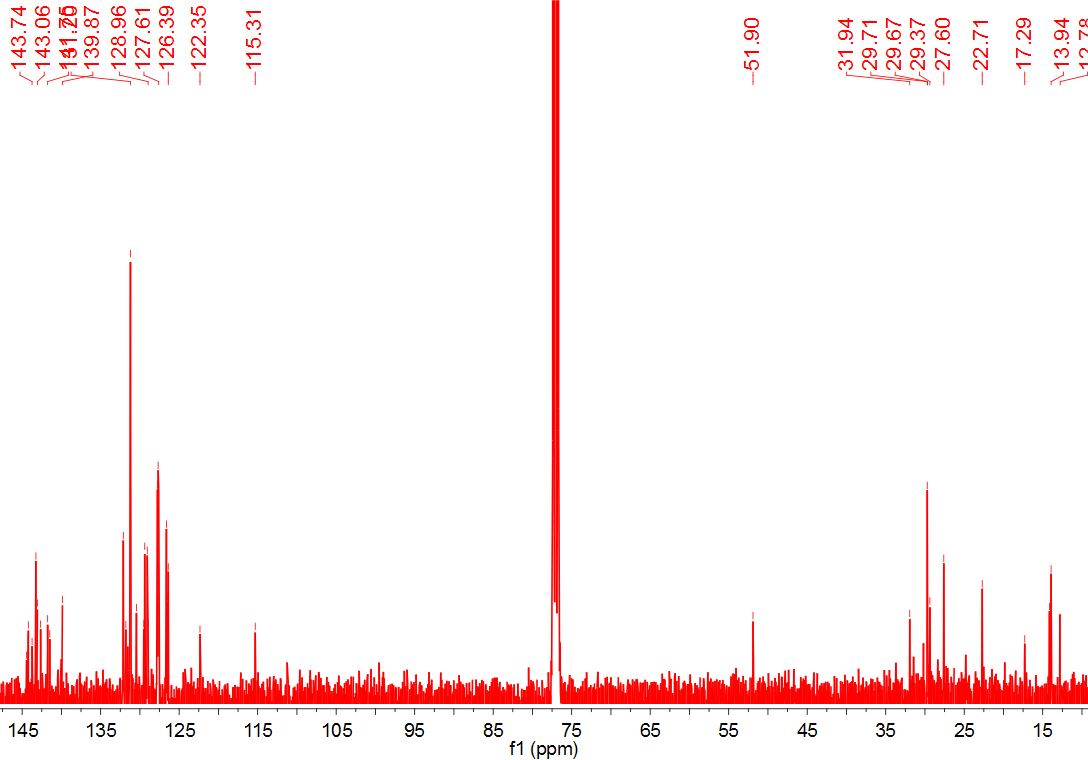


^13^C NMR spectrum for TPE-BODIPY-Cl


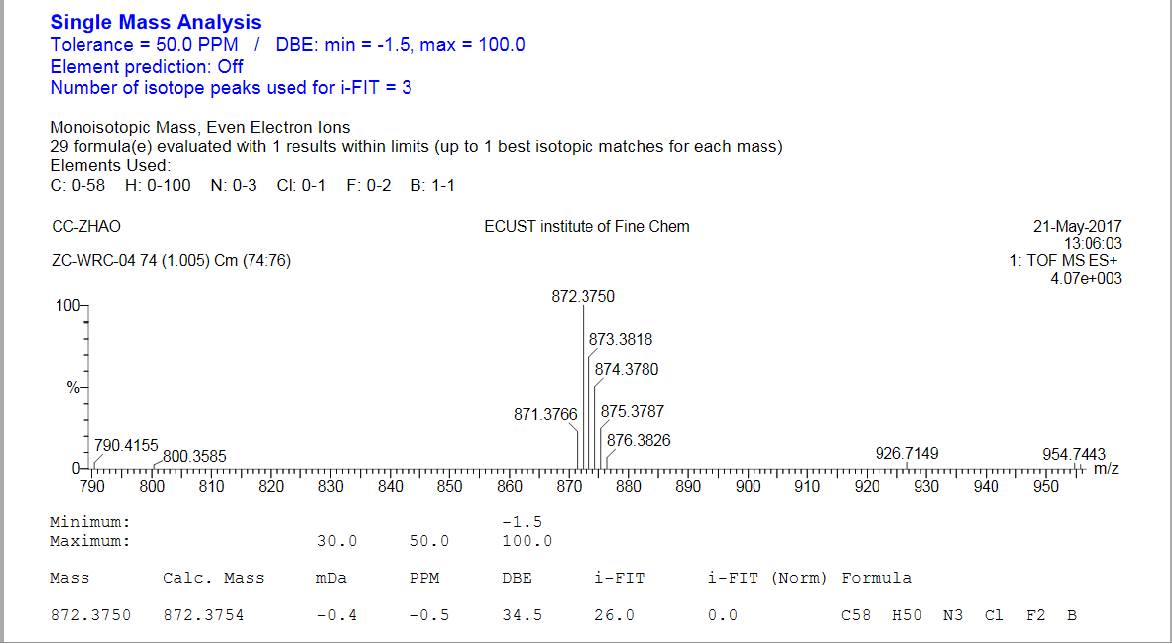


HRMS spectrum for TPE-BODIPY-Cl.
